# Advances in artificial intelligence applications for the management of chronic obstructive pulmonary disease

**DOI:** 10.3389/fmed.2025.1685254

**Published:** 2025-10-14

**Authors:** Mingyu Wang, Luhan Li, Min Feng, Zhuo Liu

**Affiliations:** The First Affiliated Hospital of Dalian Medical University, Dalian, China

**Keywords:** chronic obstructive pulmonary disease, artificial intelligence, machine learning, deep neural networks, multimodal

## Abstract

Chronic obstructive pulmonary disease (COPD), characterized by high incidence and mortality rates, is a chronic respiratory disorder that places a substantial burden on healthcare systems. Artificial Intelligence (AI), with its deep integration into the medical field, particularly through its core branches—Machine Learning (ML) and Deep Learning (DL)—has demonstrated significant potential in the intervention and management of COPD. From early risk prediction based on multimodal data to the enhancement of precise diagnosis and treatment through radiomics and clinical decision support systems, and further to the dynamic assessment of acute exacerbation and comorbidity risks via machine learning models, AI has, in combination with bioinformatics and multi-omics analysis, established a novel intelligent management framework that spans the entire disease continuum. This framework offers innovative, individualized solutions aimed at alleviating the burden on healthcare systems. This article reviews the technical applications and clinical value of AI in the diagnosis, prevention, treatment, and prognosis of COPD, discusses current challenges, and outlines future development directions to provide insights for clinical practice and research.

## 1 Introduction

Artificial Intelligence, as an emerging technology that simulates, extends, and enhances human cognitive capabilities, has become deeply integrated into the medical field across multiple dimensions. Its rapid advancement in the healthcare sector has driven innovation in medical algorithms, encompassing both disease diagnosis and treatment as well as the management of patient health data (1–3). The core branches of AI applied in the medical field include Machine Learning and Deep Learning. Machine Learning enables autonomous decision-making through the recognition of data patterns and, in clinical applications, has generated a series of diagnostic, therapeutic, and predictive models by applying AI algorithms to structured data (4–6). Deep Learning processes high-dimensional, unstructured data using multi-layer neural networks. For example, Convolutional Neural Networks (CNNs) excel in image analysis, Recurrent Neural Networks (RNNs) are effective for time-series data (such as lung sound signals), and Graph Neural Networks (GNNs) are capable of analyzing complex biological network relationships (such as airway tree structures) (7). The primary value of AI in healthcare lies in transforming raw medical data—such as images, physiological signals, and genetic information—into actionable clinical insights.

Chronic Obstructive Pulmonary Disease is a progressive respiratory disorder characterized by persistent airflow limitation. Its underlying pathophysiological mechanisms are complex, and the responses to smoking exposure and pharmacological interventions remain incompletely understood (8). As the third leading cause of mortality globally, COPD is associated with risk factors such as tobacco smoking, environmental pollutants, and genetic predisposition. Disease management primarily centers on community-based and individualized approaches. The core challenges in intervention and management include the difficulty of early diagnosis–due to reliance on specialized equipment and limited accessibility of pulmonary function testing–the high risk of acute exacerbations that may increase mortality, the presence of multiple comorbidities (such as depression and cardiovascular diseases), and substantial inter-individual variability (9, 10).

The current focus of integrating AI with respiratory medicine primarily centers on the diagnosis of chronic diseases and the development of electrochemical sensing monitoring systems (11). Machine Learning can integrate lung function assessments, transcriptomics, RNA sequencing, and imaging techniques to construct frameworks for diagnostic, predictive, and interventional models. Through standardized AI-based monitoring, it is possible to enhance lung function evaluation, achieve high-precision qualitative imaging diagnoses, and utilize Deep Learning neural networks for accurate lung sound classification to distinguish between healthy and pathological conditions, thereby enabling early detection and risk prediction of COPD (6, 12, 13). Furthermore, AI can identify COPD-related biomarkers at the molecular and genetic levels, offering more precise insights into disease progression and potential complications. The application of AI in respiratory diseases is primarily oriented toward healthcare delivery and patient self-management, particularly benefiting elderly individuals with COPD. By generating personalized management plans based on regular assessments, AI can enhance self-management effectiveness and improve treatment adherence. These interventions also contribute to alleviating the burden on healthcare systems (14, 15).

**Figure 1:**
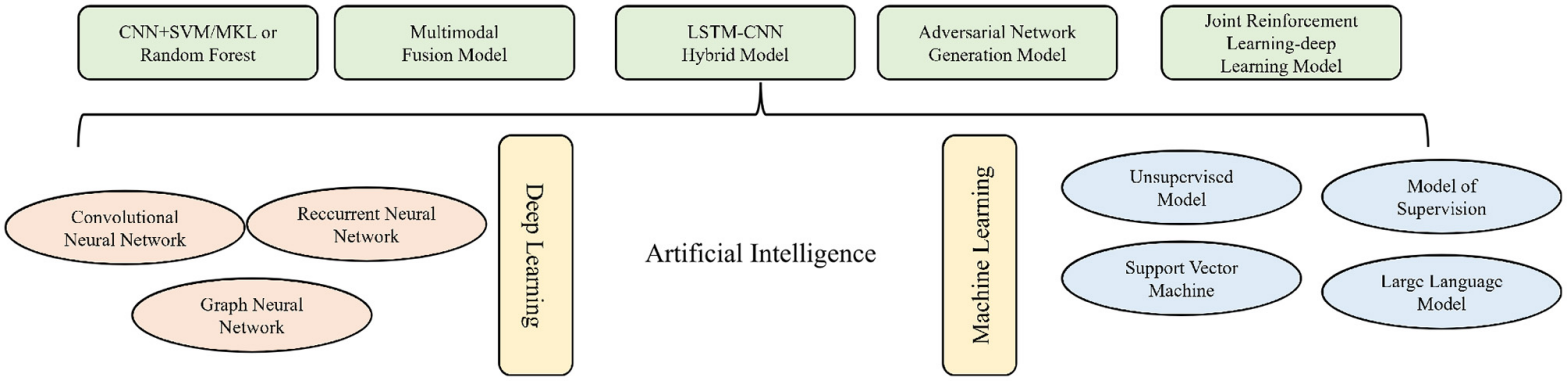
A review structure diagram of COPD and artificial intelligence.

AI is playing a pivotal role in advancing medical paradigms, demonstrating considerable potential in the early prediction, diagnostic-therapeutic optimization, and prognostic evaluation of COPD. The field encompasses a spectrum of architectures, ranging from traditional machine learning (ML), including support vector machines and random forests, to deep neural networks–such as convolutional neural networks (CNNs), recurrent neural networks (RNNs), and graph neural networks (GNNs). More recent developments include multi-modal fusion, adversarial generative models, and integrated reinforcement-learning systems, which substantially improve capabilities in processing and interpreting multi-source, heterogeneous medical data. To provide a systematic overview of the AI models discussed herein and their applications in COPD management, we have categorized key algorithms and their respective functions in Figure 1. This figure serves as a conceptual and technical framework for the subsequent discussion of each model's mechanistic role and clinical utility across the stages of prediction, diagnosis, and prognosis in COPD. Finally, this review systematically examines the role of AI in predictive and early-warning systems, innovations in diagnostic identification, and the distinctive implications for prognostic assessment in COPD.

## 2 The role of artificial intelligence in the early prediction and warning of COPD

The continuous refinement of various intelligent, data-driven models within the medical field significantly enhances the precision of early screening and predictive capabilities for patients with COPD. The integration of AI improves both the accuracy and efficiency of diagnostic assessments. Furthermore, intelligent predictive systems enable the rapid identification of abnormal indicators, support clinical decision-making processes, and hold the potential to uncover novel biomarkers and disease patterns. These systems are distinguished by their user-friendly design and cost-effectiveness, thereby making them highly suitable for broad implementation across primary healthcare settings.

In the prevention of COPD, AI algorithms are primarily integrated into various digital health platforms, focusing on processing complex and high-dimensional data generated by sensors, wearable devices, and other health monitoring technologies. These raw health data are transformed into actionable insights, thereby achieving the goal of early high-precision prediction (16). In resource-limited primary healthcare settings, specialized recording programs designed with neural networks, i.e., deep learning methods, can be applied to accurately identify lung sounds through exhalation and coughing, further predicting the risk of COPD (13, 17). Research indicates that the LASSO model, based on machine learning and incorporating various algorithms and random forest models, may be the optimal risk model for screening potential COPD cases. This model can also predict potential susceptibility loci, enabling early identification of high-risk populations and facilitating personalized health interventions to delay or prevent disease onset (18). Currently, a clinical prediction model for COPD has been developed using high-dimensional weighted gene co-expression network analysis combined with ML and RNA sequencing data. ML is particularly sensitive in predicting cardiopulmonary dysfunction and related diseases in the elderly (15). In regions with relatively accessible medical resources, ML can be linked with CT imaging to predict the likelihood of high-risk individuals progressing to COPD (19).

The rapid advancement of AI has markedly improved the accuracy of predicting COPD using limited clinical data and pulmonary function measurements, while also reducing inter-physician variability in subjective assessments. For example, by analyzing recordings of the vowel “a” or signals from accelerometers placed at lung auscultation sites, AI systems can achieve high sensitivity and specificity in COPD prediction based on features such as pronunciation, breathing patterns, and respiratory rate. Particularly in regions with constrained medical resources, AI-assisted respiratory disease evaluation holds promise for expanding access to high-quality medical care (20–22).

Despite the potential of sound-based AI models in the early detection of COPD, several limitations impede their current clinical applicability. Firstly, the generalizability of these models remains limited: most existing studies (23, 24) rely on single-center, small-sample datasets that underrepresent rare disease subtypes and specific populations (e.g., pediatric patients), and are vulnerable to inconsistencies in recording conditions, thereby hindering broader population-wide deployment. Secondly, the translation of acoustic signals into physiologically meaningful parameters poses theoretical challenges—specifically (25), the nonlinear relationship between smartphone-captured sound amplitude and expiratory flow rate remains poorly characterized, and oversimplified processing approaches may introduce systematic bias. Furthermore, most models (26) are currently restricted to binary classification (e.g., “healthy” vs. “unhealthy”), lacking discriminative capacity for differentiating among specific respiratory diseases such as COPD and asthma, which limits their utility in clinical decision-making for precise diagnosis. Additionally, many algorithms remain at a proof-of-concept stage, with performance benchmarks still at baseline levels and without rigorous validation against spirometry as the gold standard.

To overcome these challenges, future efforts should prioritize the development of large-scale, multi-center, standardized prospective cohorts that incorporate multidimensional clinical variables such as smoking history and genetic predisposition to enhance model robustness. Technologically, further investigation into advanced neural network architectures—including convolutional and time-series models—is warranted. The development of interpretable AI approaches could help elucidate the mechanistic links between acoustic features and airway pathophysiology. Ultimately, through combined software and hardware innovations (e.g., high-fidelity recording devices) and prospective clinical validation, such models may transition from experimental tools to practical applications—particularly in primary care and home-based health monitoring—enabling early screening and effective of COPD.

## 3 Optimization and innovation of artificial intelligence in the diagnosis and treatment of chronic obstructive pulmonary disease

The integration of AI algorithms into digital health management for COPD enables smartphones and AI-powered devices to objectively capture respiratory symptoms, while digital technologies can monitor changes in physiological and behavioral states (15). In the diagnostic domain, artificial intelligence has been explored through auscultation, pulmonary function tests, and imaging for diagnostic and phenotypic analysis. The multidimensional optimization of AI plays a pivotal role in clinical diagnosis and treatment, which will transform the entire patient care pathway from diagnosis to symptom alleviation.

The current application of AI in respiratory systems involves the utilization of wearable devices, intelligent equipment, and medical sensors to collect measurable and quantifiable physiological, behavioral, and environmental parameters of individuals (27–29). By leveraging digital technologies, physicians can gather data and AI insights, such as interpreting diagnostic genes for COPD in immune cell subsets through ML algorithms, and constructing clinical diagnostic models. Concurrently, the stability of these diagnostic models in predicting chronic obstructive pulmonary disease is evaluated using AUC curves in conjunction with bulk RNA sequencing data (30, 31). The analysis of respiratory sound recordings (cough and breath) using ML-based monitoring technologies, combined with convolutional long short-term memory networks, Mel-frequency cepstral coefficients, and chromatograms to capture relevant acoustic features, represents a newly developed edge computing system for the automatic detection of chronic respiratory diseases based on audio analysis. This system exhibits high sensitivity and specificity, facilitating the diagnosis of COPD by primary healthcare workers solely through respiratory sounds (32–34).

In the field of medical imaging, artificial intelligence has transcended the limitations of traditional visual assessment through the application of high-precision segmentation and quantification techniques in computed tomography, offering a promising pathway for the transformation of chronic obstructive pulmonary disease diagnosis and management. Deep learning technology has demonstrated substantial potential in the medical imaging analysis of COPD, particularly in conjunction with CT, optimizing radiological diagnosis through both qualitative and quantitative enhancements (35–38). By analyzing key radiological features in CT, such as airway alterations, emphysema, and vascular characteristics, DL has improved diagnostic accuracy and efficiency through CNN-based lobar and airway tree segmentation and ventilation quantification, thereby providing more precise treatment strategies for COPD patients (39–43). Furthermore, the integration of multimodal models combining CT imaging, spirometry data, and demographic characteristics has elevated COPD diagnostic accuracy to over 90%, surpassing single indicators. The application of deep learning convolutional neural networks for automated staging of COPD patient CT scans has enabled the prediction of disease progression and mortality (44–46).

Furthermore, AI, in conjunction with multi-omics databases, has comprehensively explored COPD-specific biomarkers from genes to proteins across multiple dimensions. Literature indicates that machine learning algorithms, integrated with bioinformatics analysis, can prioritize key biomarkers of COPD, thereby providing insights into potential therapeutic targets (47). ML also facilitates the identification and computational analysis of COPD microarray datasets, offering diagnostic markers and personalized immunotherapy targets by delineating aberrant immune cell profiles associated with the disease (48). The introduction of digital biomarker monitoring has enabled the decentralization and widespread adoption of COPD diagnostic and therapeutic technologies, creating significant opportunities for primary and secondary healthcare. This advancement supports health risk assessment and the prevention of disease progression in vulnerable sub-healthy populations through continuous monitoring of relevant health parameters via wearable devices or smartphone applications, allowing both patients and clinicians to track COPD progression in real-time (49).

Although the application of artificial intelligence in COPD diagnosis and treatment continues to advance, the clinical applicability of AI models remains limited by several persistent challenges. First, the performance of image analysis algorithms is highly dependent on standardized image acquisition (50). For instance, in expiratory-phase CT imaging, suboptimal patient cooperation may introduce motion artifacts or reconstruction inaccuracies, compromising the quantitative assessment of air trapping. Second, most existing models (51) focus primarily on quantifying emphysema and air trapping, yet fail to adequately incorporate key airway structural parameters—such as airway wall thickness and lumen diameter—thereby constraining their ability to fully characterize heterogeneous COPD phenotypes. Furthermore, diagnostic models (52) based on biomarkers or imaging features are susceptible to clinical heterogeneity, including variations in population baseline characteristics, sampling protocols, and experimental conditions, which diminishes model robustness and consistency across cohorts. In the analysis of the immune microenvironment, computational approaches like transcriptomic deconvolution currently lack the resolution to accurately distinguish cell subtypes, and their outputs still require validation through wet laboratory experiments.

To address these limitations, future efforts should prioritize the establishment of multi-center standardized imaging databases and the development of AI tools capable of automatically detecting image quality issues and acquisition artifacts. Concurrently, integrated modeling frameworks should be developed that incorporate multidimensional data—including airway morphology, extent of emphysema and air trapping, serum biomarkers, and clinical features. To mitigate clinical and technical heterogeneity, strategies such as transfer learning and domain adaptation should be employed to enhance model generalizability, complemented by explainable AI techniques to improve the interpretability and clinical credibility of predictions. At the mechanistic level, expanding the use of spatial transcriptomics and single-cell sequencing data to validate computational inferences is recommended, thereby facilitating the translation and practical application of AI in precision diagnosis and management of COPD.

## 4 Artificial intelligence in prognostic evaluation of chronic obstructive pulmonary disease

The prognosis of patients with COPD is primarily determined by the occurrence of acute exacerbations and the presence of comorbidities. Machine learning and deep learning-based artificial intelligence (AI) models for long-term prognostic prediction in COPD patients can aid both clinicians and patients in understanding potential disease progression and implementing timely interventions to mitigate adverse outcomes (6, 45, 53, 54). Moreover, the onset of acute exacerbations of COPD may intensify the clinical manifestations of existing comorbid conditions.

Existing research has confirmed that the ML-enhanced CatBoost model, when integrated with features extracted from electronic stethoscopes (such as maximum/minimum vibration energy), has demonstrated efficacy in predicting AECOPD events. These models hold promise for enhancing remote monitoring, enabling early risk assessment, and providing a basis for treatment decisions in home-based COPD patients (55). The improved DDRIME algorithm can estimate the risk of acute exacerbation requiring mechanical ventilation, exhibiting high accuracy and facilitating early intervention in AECOPD patients, thereby effectively reducing morbidity and mortality (56, 57). The application of ML methods to integrated data analysis in COPD patients, predicting impending disease exacerbation models, contributes to improved disease management and supports proactive interventions to minimize disease progression (58–60). Certain AI-supported systematic reviews provide evidence for identifying markers of acute heart failure in COPD patients, such as interpreting BNP in conjunction with imaging and clinical signs, incorporating the potential for acute heart failure exacerbation in COPD patients (47, 61). Digital inhalers can enhance medication adherence and control, while symptom tracking, pulmonary function monitoring, and environmental parameters aid in identifying triggers for disease exacerbation. In assessing disease remission, digital technology can capture the majority of the defined aspects of remission, from disease exacerbation to the use of remission medications (62).

Current derivative predictive models for comorbidities in COPD primarily focus on patients with concomitant depression, airway obstructive mucus plugs, and lung cancer. Research has demonstrated that the prevalence of depression in patients with stable chronic obstructive pulmonary disease ranges from 10% to 42%, potentially exceeding 80% during acute exacerbations, significantly compromising the quality of life for COPD patients. Consequently, the derivative predictive algorithm model serves as a streamlined online platform, enabling healthcare professionals to utilize ML algorithms for identifying COPD patients at risk of depression anytime and anywhere, thereby facilitating early detection and timely intervention (63). The integration of circulating tumor DNA detection with ML models in COPD patients enables precise prediction of ctDNA mutation carriers, allowing for specific identification of COPD patients with concomitant lung cancer, with the ultimate goal of achieving early diagnosis and proactive treatment.

The application of artificial intelligence in COPD prognosis assessment continues to face challenges related to model construction and clinical translation. Most existing prediction models (64) are developed using single-center, retrospective data with limited sample sizes and a low number of clinical events, significantly compromising their generalizability and stability. Furthermore, these models (65) often exhibit limited interpretability, high computational complexity, sensitivity to hyperparameter settings, and dependence on manual feature engineering, which collectively restrict their adaptability and clinical credibility across heterogeneous medical datasets. In terms of prognostic evaluation, current models predominantly rely on static or single-modal data, lacking effective integration of multidimensional temporal information such as dynamic physiological parameters, real-time treatment adherence, and environmental factors. This renders them vulnerable to recall bias and data incompleteness, thereby reducing predictive accuracy. Moreover, most models have not undergone multi-center external validation nor have they been deeply embedded within routine clinical workflows. Their real-world utility and capacity to improve patient outcomes remain to be substantiated through prospective interventional studies.

To enhance the practical utility of AI in COPD prognosis, it is essential to establish large-scale, multi-center prospective cohorts and to develop interpretable, lightweight algorithmic frameworks capable of integrating multimodal dynamic data. Additionally, efforts should focus on strengthening the integration of AI systems with clinical information systems—such as electronic medical records and mobile monitoring devices—to build prognostic tools equipped with temporal awareness and personalized adaptability. The clinical benefit and broader applicability of such tools must be evaluated in real-world settings.

## 5 Discussion

In the progression and prognosis of COPD, AI has developed a “multi-algorithm, multi-dimensional” COPD management system by integrating multimodal data from imaging, genomics, microbiomics, and provincial health databases. Table 1 presents an overview of the application of AI in COPD management. This system embeds lightweight algorithms into wearable devices through edge intelligence, enabling real-time data processing and immediate intervention, thereby reducing reliance on cloud computing. Through policy coordination, an AI model registration and regulatory framework has been established, promoting the inclusion of “real-world evidence” into clinical guidelines, such as the FDA's approval standards for AI functionalities in digital inhalers.

**Table 1:** Applications of AI in COPD management.

**Clinical application**	**Model**	**Data sources**	**Advantages**	**Limitations**
Early prediction and warning	LASSO, Random Forest, Ensemble Learning Models	Breath sounds, cough sounds, speech, lung function, CT imaging, pathological tissue, RNA sequencing. Multimodal approaches: image + speech, image + text	Early identification of high-risk populations; non-invasive; high flexibility and sensitivity	Insufficient data standardization, limited sample size, poor generalizability
Diagnosis and treatment	CNN (image segmentation), CNN+LSTM (pulmonary nodule), GNN (airway structure), multimodal fusion models	CT/X-ray, lung sounds, demographic information, electronic medical records, biomarkers. Multimodal approaches: image + text, image + voice, speech recognition + App	Automated diagnosis, reduced human error; combining imaging and clinical features improves accuracy; supports individualized medication and disease management	Deep learning has interpretability issues, complex multimodal construction, poor generalizability and local adaptability
Prognosis evaluation	CatBoost (AECOPD prediction), DDRIME (mechanical ventilation demand), deep learning (survival prediction), ctDNA+ML (lung cancer comorbidity risk)	Electronic auscultation, lung function, CT imaging, liquid biopsy biomarkers. Multimodal approaches: image + clinical + genetic	Enables prediction of disease progression, exacerbation risk, and mortality; assists personalized management and resource allocation	“Black box” problem of models affecting clinical trust; lack of long-term follow-up data; supervision and privacy issues

According to reports, a novel machine learning tool for “subtype and stage inference” was already in existence by 2020. This tool can identify COPD patient subtypes with distinct longitudinal progression patterns through “tissue-to-airway” and “airway-to-tissue” subtype recognition, providing a new imaging biomarker for early disease classification and staging (66). In respiratory diseases, deep learning approaches combined with neural network analysis can estimate total lung volume from pixel-level thickness maps of chest radiographs, thereby assessing disease severity (67, 68). The newly discovered AI-enhanced model integrates clinical variables (medical history, dyspnea, and inhalation therapy) with spirometry image data (flow-volume loops and volume-time curves) to accurately predict the likelihood of moderate-to-severe and severe exacerbation events within one year (69). Currently, the AutoCOPD screening model developed by Academician Zhong Nanshan's team in China, based on quantitative CT of the full lung during inspiration, can accurately identify COPD using only 10 QCT features (70). Internationally, deep learning models combined with chest radiographs can also predict survival rates in COPD patients (71). AI can further provide personalized treatment recommendations for physicians by analyzing individual patient characteristics, such as medication dosage and rehabilitation training plans. However, the limited interpretability of AI diagnostic results poses challenges for clinicians to fully comprehend the model's decision-making basis, affecting trust in clinical applications. Subsequent research should focus on enhancing explainable AI, developing visualization tools to demonstrate key decision nodes and data evidence in the diagnostic process, thereby improving physician acceptance of AI diagnostic outcomes.

International research has explored the inference of chronic obstructive pulmonary disease (COPD) through deep learning of raw spirometry graphs, identifying novel genetic loci and improving risk models, which facilitates early intervention at the disease's incipient stage to delay its progression (71). Artificial intelligence (AI) can also be integrated with Mendelian randomization analysis to predict inflammatory responses and ferroptosis-related immune cell and gene signatures in pre-COPD patients, thereby advancing the development of targeted traditional Chinese medicine (TCM) for COPD-related biomarker screening and efficacy prediction (72). However, current AI data collection faces limitations, with inconsistent formats and standards across different regional sources, impacting the comprehensiveness and accuracy of model training. Future efforts should establish unified data collection standards, reduce variability in diagnostic judgments, expand sample sizes to include data from more regions, ethnicities, and disease stages, and enhance the generalization capability of predictive models. Additionally, current assessment models for COPD's complex comorbidities remain inadequate, with intricate interrelationships among different comorbidities posing challenges to AI model construction. Subsequent research should focus on developing comprehensive multi-comorbidity assessment models, delving into disease interaction mechanisms, targeted drug efficacy prediction, and incorporating more prognostic factors such as patients' living environments and psychological states. This will enable more comprehensive and precise prognostic evaluations, providing stronger support for personalized treatment strategies.

In summary, artificial intelligence presents innovative opportunities for personalized intervention management in COPD. By analyzing multi-source data including imaging, pulmonary respiratory factors, and genetic information, AI has revolutionized the entire COPD management process, encompassing diagnosis (e.g., CT-based precise quantification), prevention (e.g., home-based risk screening), treatment (e.g., personalized medication), and prognosis (e.g., exacerbation prediction). Despite challenges in data integration, algorithm development, and clinical implementation, with technological iterations and enhanced interdisciplinary collaboration, AI is poised to facilitate the transformation of COPD management from “passive treatment” to “active health” and advance precision-targeted drug development, thereby providing sustainable solutions for global respiratory health.
